# A Sorrow Shared Is a Sorrow Halved: Moral Judgments of Harm to Single versus Multiple Victims

**DOI:** 10.3389/fpsyg.2016.01142

**Published:** 2016-08-02

**Authors:** Daffie Konis, Uriel Haran, Kelly Saporta, Shahar Ayal

**Affiliations:** ^1^School of Psychological Sciences, Tel Aviv UniversityTel Aviv, Israel; ^2^Department of Management, Ben-Gurion University of the NegevBeer-Sheva, Israel; ^3^Department of Psychology, The Open University of IsraelRaanana, Israel; ^4^Baruch Ivcher School of psychology, Interdisciplinary CenterHerzliya, Israel

**Keywords:** moral judgments, deception, individual victims, multiple victims, identifiability, attribution

## Abstract

We describe a bias in moral judgment in which the mere existence of other victims reduces assessments of the harm suffered by each harmed individual. Three experiments support the seemingly paradoxical relationship between the number of harmed individuals and the perceived severity of the harming act. In Experiment 1a, participants expressed lower punitive intentions toward a perpetrator of an unethical act that hurt multiple people and assigned lower monetary compensation to each victim than did those who judged a similar act that harmed only one person. In Experiment 1b, participants displayed greater emotional involvement in the case of a single victim than when there were multiple victims, regardless of whether the victims were unrelated and unaware of each other or constituted a group. Experiment 2 measured the responses of the victims themselves. Participants received false performance feedback on a task before being informed that they had been deceived. Victims who were deceived alone reported more negative feelings and judged the deception as more immoral than did those who knew that others had been deceived as well. Taken together, these results suggest that a victim’s plight is perceived as less severe when others share it, and this bias is common to both third-party judges and victims.

## Introduction

The CNBC television series CNBC’s “American Greed” (2007) tells the story of Barry Hunt who solicited money from several people across the United States by presenting them with phony investment opportunities and promising them high returns only to disappear with their savings. The show presents Elaine Nelson, one of Hunt’s victims, who was left penniless after having invested all of her money in one of Hunt’s companies. When contacted by FBI agents, Ms. Nelson was told about Hunt’s other successful con operations. Should the knowledge that there are additional victims increase Barry Hunt’s moral liability for his actions? From both a legal and a normative perspective, the answer is undoubtedly “yes.” When all else is equal, a crime against several people causes more overall harm than a similar crime against one person, and should carry more severe punishment as is the case in the criminal code. Theoretical models of ethical judgment take a similar view. According to the Moral Intensity Model (Jones, 1991, p. 374), “an act that causes 1,000 people to suffer a particular injury is of greater magnitude of consequence than an act that causes 10 people to suffer the same injury.”

However, from a psychological perspective, people may not adhere to this normative principle. Although moral judgments of harmful acts are greatly influenced by assessments of the harm they caused (e.g., Baron and Hershey, 1988; Mazzocco et al., 2004; Gino et al., 2009), the relationship between the two is not monotonic. This is particularly evident with regard to the number of victims. Research has repeatedly found that numerosity has a significant negative impact on people’s affective reactions. For example, life-saving interventions are often perceived as more beneficial when fewer lives are at risk (Fetherstonhaugh et al., 1997). Similarly, a single suffering victim produces a stronger emotional involvement than many victims (Cameron and Payne, 2011; Kogut, 2011), and the existence of more victims is associated with *lower* willingness to donate money for the victim’s (or victims’) cause. For example, Västfjäll, Peters and Slovic (see Slovic, 2007) gave a group of participants the opportunity to donate money to help one starving child in Africa and another group the opportunity to help a similar child in distress. A third group was given the opportunity to help both children. Participants’ affective reactions, as well as the amounts donated, were significantly lower in the combined condition than in any of the individual-victim conditions. This effect is not restricted to life-threatening situations: in another study, participants judged a financial fraud as more severe and thus deserving of more severe punishment when it harmed three victims than when it harmed a group of thirty (Nordgren and McDonnell, 2010).

Nordgren and McDonnell (2010) explained their findings in terms of the identifiable victim effect (e.g., Small and Loewenstein, 2003; Small et al., 2007). Briefly, this effect refers to people’s greater sympathy and willingness to help personalized, single victims (who are presented with some information that distinguishes them from others) than “statistical victims” who are perceived as a faceless mass. Although the classic studies on this effect employed completely different dependent measures (e.g., helping behavior, willingness to donate) and did not test the effect of number of victims directly, they propose a reasonable explanation for the greater impact of a single identifiable victim in terms of both affective reactions (i.e., heightened compassion and sympathy) and generosity (i.e., greater willingness to donate). Nordgren and McDonnell (2010) argued that victim identifiability is also a central mechanism underlying judgments of intentionally harmful acts. For instance, they demonstrated that increasing the identifiability of one member of the victim group attenuates the effect of group size on judgments of harm.

Note that the studies cited above examined the participants’ affective reactions and/or willingness to help from a bystander’s perspective. Their judgments may be fundamentally different from those of the victim of a harmful act. For example, the victim may materially benefit from the presence of other victims who share her plight by receiving social support and an opportunity to join forces. As a result, the perceived harm caused by the transgression to each individual victim may be alleviated, leading to more forgiving judgments than what the normative approaches would suggest.

This study was designed to extend previous findings of the number of harmed individuals on people’s moral judgment in several ways. First, we examined this effect in everyday situations where the victim was in no physical danger. Second, whereas most studies have measured people’s willingness to actively help victims, we examined the participants’ moral judgments and assessments of the victims’ suffering independently of any need to take action. Third, whereas earlier work has focused on judgments by third party participants who were uninvolved in the situation, no study, to the best of our knowledge, has examined the judgments and reactions of the victims themselves. Here, we examined whether individuals who were harmed would perceive their own suffering as less severe when they were informed that others had been harmed as well. Finally, we probed previous arguments that have associated victim numerosity with identifiability. By measuring the victims’ own reactions, we were able to manipulate group size while ensuring that the targets were equally identifiable in both single and multiple victim conditions.

We report results from three experiments. Experiments 1a and 1b used hypothetical scenarios that varied the number of victims. These experiments examined people’s harm assessment and emotions from a bystander’s point of view. In Experiment 2, the actual victims of the harmful act were the participants themselves. Following the realization of their transgression, we measured participants’ feelings and attitudes toward that act. We hypothesized that across all three studies, a transgression would be perceived as less harmful and less immoral when it affected multiple individuals than when it harmed only one.

## Experiment 1a

Experiment 1a tested the effect of multiple victims on evaluations of a transgression, both with regard to the harm suffered by each victim and the overall severity of the act. In a three-group design, we presented participants with a scenario describing a harmful act and varied the number of individuals affected by it. We then measured participants’ judgments and attitudes toward the act.

### Method

#### Participants

One hundred twenty-seven members of a U.S. university’s online participant pool (84 female, Mean age = 34.10 years, *SD* = 12.08) were recruited to participate in a web-based study for a 1 in 10 chance of winning a $10 Amazon gift card. Participants signed an online consent form at the beginning of the experiment, and were debriefed at the end.

#### Procedure

The experiment used a three-group design. In each condition, participants read a hypothetical story describing Dr. Stillman, a dentist and an avid angler, who is invited to go on a long weekend fishing trip and decides to reschedule patients who are in pain and waiting for treatment. Each participant read one of three versions of the scenario, which were all identical except for the number of patients who were affected by the dentist’s trip. Participants in one condition read about one patient, another about three patients, whereas in the third condition, participants read about 10 patients whose appointments were postponed at the last minute by the dentist.

After reading the scenario, participants rated their attitudes toward the behavior described in the scenario. Specifically, they assessed the severity of the harm suffered by an individual victim by estimating the smallest monetary amount they would consider a sufficient financial compensation for each victim. Participants also rated their support for suspending the dentist’s license to practice dental work and for filing a civil lawsuit against him. These three items were presented in a random order. Finally, we measured participants’ emotional reactions by asking them to indicate the extent to which they think the patients felt hurt by the dentist’s act, the extent to which they perceived the dentist as guilty for the patients’ suffering, and their sympathy for the victim(s). Participants’ emotional responses were measured on a continuous scale ranging from 0 (no emotion at all) to 10 (the strongest possible emotion).

### Results

We conducted a one-way ANOVA to measure the effect of the total number of victims on participants’ estimates of the lowest sufficient compensation for an individual victim. The analysis revealed a significant main effect for the number of victims [*F*(2,124) = 4.45, *p* = 0.01, $\eta_{p}^{2}$ = 0.07]. A planned contrast between the single-victim condition and the two multiple -victim conditions found that the minimal sufficient compensation per victim was about twice the estimated amount when there was only one harmed individual compared to three or ten victims [*t*(124) = 2.90, *p* = 0.004, *d* = 0.52]. When there were three versus ten victims, there was no significant difference in the minimal compensation [*t*(124) = -0.99, *p* = 0.32, *d* = -0.18] (See **Table 1**).

**Table 1:** Perceptions of deserved compensation for each individual victim and punishability of the act (Means and SD’s), by the total number of victims in Experiment 1a.

Total number of victims	Compensation for the individual victim (*SD*)	Willingness to instigate sanctions against the agent (*SD*)
1	2561.84 (3172.66)	5.72 (3.09)
3	981.06 (1075.53)	3.23 (2.66)
10	1444.38 (1907.03)	4.15 (2.81)


The two measures of participants’ attitudes toward a suspension of the dentist’s license and a malpractice lawsuit against him were highly correlated (*r* = 0.72); therefore, we calculated the average of the two for each participant and conducted a one-way ANOVA on participants’ support for these sanctions. As shown in **Table 1**, participants were more supportive of sanctions against the dentist when fewer victims were harmed by his behavior [*F*(2,124) = 6.18, *p* = 0.003, $\eta_{p}^{2}$ = 0.09]. A planned contrast between the single victim condition and the multiple victim conditions confirmed our main hypothesis [*t*(124) = 3.46, *p* = 0.001, *d* = 0.62]: participants were significantly more willing to instigate sanctions against a perpetrator who had harmed a single victim than against a person who had harmed several victims. No significant difference was found between the two multiple victim conditions [*t*(124) = -1.37, *p* = 0.17, *d* = -0.24]. We also found no significant intergroup difference for the emotional reaction items (All *Fs* < 1, see **Table 2**).

**Table 2:** Emotional reactions (Means and SD’s) by the total number of harmed patients in Experiment 1a.

Total # of harmed patients	Sadness (*SD*)	Anger (*SD*)	Sympathy (*SD*)	Guilt (*SD*)	Feeling hurt (*SD*)
1	4.14 (3.12)	5.63 (3.08)	7.46 (2.31)	7.00 (2.61)	6.09 (2.56)
3	4.27 (3.20)	5.60 (2.41)	7.38 (1.72)	7.25 (2.27)	6.20 (2.10)
10	4.08 (2.62)	5.39 (2.51)	6.98 (2.30)	7.20 (2.26)	6.11 (2.07)


*The scale ranges from 0 (weakest) to 10 (strongest).*

### Discussion

Experiment 1a found that the mere existence of additional victims affected people’s assessments of an unethical act. A patient was perceived to be deserving of less compensation when other patients were harmed by the dentist’s act than when she was a single victim. This bias in participants’ perceptions was strong enough to affect their perceptions of the overall severity of the act: when several patients suffered (either three or ten), participants’ support for instigating sanctions on the agent *decreased* rather than increased.

However, a few caveats should be considered. First, affective reactions to the dentist’s transgression were not significantly different between conditions. This may suggest that emotional reactions and psychophysical numbing did not play a significant role in determining the effect on moral judgment or punitive motives. Alternatively, the order in which we administered the questionnaire items may have attenuated the emotional effect. Since we measured the participants’ affective reactions *after* measuring their willingness to prosecute the dentist and compensate his patients, the consideration of punitive actions may have relieved some of the negative feelings (c.f., Ayal and Gino, 2011; Barkan et al., 2012). In Experiment 1b we focused on participants’ affective reactions, and measured them immediately after manipulating the number of victims.

Experiment 1b addressed two other alternative explanations for Study 1a. First, in the two multiple victim conditions, participants could have perceived the victims as an entitative, communicating group. In such settings group members can provide support to each other, which is not available to a single victim. Such group support may *actually* alleviate the victim’s suffering, and this in turn may reduce punitive intentions in these settings. Second, it could be argued that the harm in the single victim case was more easily avoidable. When there was only one patient waiting, the dentist could have easily treated this patient and still been able to go on the trip, but having many appointments forced the dentist to choose between moving all the appointments or canceling the trip altogether. Therefore, harming a single victim may be less harmful overall, but also more easily avoidable and therefore deserving of harsher punishment. In Experiment 1b we addressed these concerns.

## Experiment 1b

Similar to Experiments 1a and 1b was based on a hypothetical scenario, albeit in a context which is more frequently associated with unethical acts: financial consulting. The victims in this story were clients of a financial advisor whose unprofessional, negligent behavior caused substantial losses to their retirement savings. In a three-group design, we varied the number and organization of the victims of the financial advisor’s negligence. The scenarios were constructed to have a single victim, five unrelated victims (who had no contact with or awareness of each other), or a group of five clients who had a joint portfolio which the financial advisor handled. The two multiple victim conditions were designed to rectify two caveats related to Experiment 1a. The first was the mere existence of multiple victims and the victims’ subjective experience in dealing with harm alone or in a group. The second was the number of victims as a function of the ease with which the harm could be avoided by the transgressor.

### Method

#### Participants

Ninety-one members of a U.S. university’s online participant pool (54 Female, Mean age = 35.5 years, *SD* = 13.2) participated in this experiment in exchange for a 1 in 10 chance of winning a $10 Amazon gift card. Participants signed an online consent form at the beginning of the experiment and were debriefed at the end.

#### Procedure

Participants completed the study online. The experiment began by reading a story about a financial advisor named Jeff who specializes in investments for retirement plans. One day Jeff reads an analyst’s report that forecasts a sharp drop in the market and strongly recommends selling S&P 500 index stocks as quickly as possible. Despite placing this recommendation at the top of his to-do list, Jeff leaves work early to meet a friend, without touching his clients’ portfolios. The next morning the stock market falls and each client whose portfolio had index shares lost $2,500 of the value of the stock. We specified that each client had $20,000 worth of S&P 500 shares the previous day, and that this value declined to $17,500. One version of the story included one such client, another presented five unrelated clients and the third was about a group of five clients who had opened a joint plan together, managed by Jeff.

After reading the story, participants evaluated the victims’ experience of the consequences of Jeff’s behavior. They were asked to refer to the experience of each client and rate the extent to which the client was hurt by the advisor’s behavior, the extent to which trust was violated between the client and the advisor, the level of guilt the advisor should feel and how bad they would feel if they were in the client’s shoes. These responses were measured on a continuous scale ranging from 0 to 10, in which the lowest and highest points were specified (e.g., 0 = “Not hurt at all”; 10 = “More hurt than he will ever be in his life”). The order of presentation of the questions was randomized.

### Results

The judgment items of the perceived harm, the extent to which trust was violated, and the perceived guilt of the transgressor had high inter-item reliability (α = 0.84). Therefore, we calculated the means of these three items. We conducted a one-way ANOVA on this combined factor to measure the effect of the total number of victims on participants’ assessments. The ANOVA revealed a significant main effect for the number of victims [*F*(2,89) = 3.15, *p* = 0.048, $\eta_{p}^{2}$ = 0.07]. The first planned contrast, which tested whether the perceived suffering of an individual victim decreased when other victims existed, revealed a significant difference between the single victim condition and the multiple victim conditions. Participants’ emotional responses in the *individual victim* condition were significantly more extreme (*M* = 6.34, *SD* = 1.25) than in the *multiple-victim* groups [(*M* = 5.63, *SD* = 1.40), *t*(89) = 2.39, *p* = 0.02, *d* = 0.54]. The second contrast, which tested whether each victim’s suffering was alleviated by being part of an entitative group, revealed no significant difference between the *five isolated victims* condition and the *group of victims* condition [*t*(89) = -0.51, *p* = 0.61, *d* = 0.25]. **Table 3** lists the group means and standard deviations of these measures.

**Table 3:** Emotional responses, by the total number of harmed clients in Experiment 1b.

# of clients affected by Jeff’s action	Mean emotional response (*SD*)
Single client	6.34 (1.25)
5 isolated clients	5.55 (1.22)
5 connected clients	5.91 (1.63)


*The scale ranges from 0 (weakest) to 10 (strongest).*

### Discussion

Experiment 1b replicated and expanded the findings of Experiment 1a. This experiment focused on participants’ affective reactions, and measured them immediately after administering the number of victims manipulation. Consistent with our hypothesis, the mere existence of additional harmed individuals *decreased* the perceived harm suffered by each individual. A loss of $2,500 was considered more painful when the victim of the loss was the only one, compared to when there were other people who also lost $2,500 each, regardless of whether the group members were in contact or not. Similarly, the participants judged the investment counselor to bear more *guilt* (rather than less guilt) when a single client was affected by his misbehavior.

Experiment 1b helps to refute the possibility that participants may have recognized some implied advantages to situations where several victims are involved, such as getting social support from other victims. In Experiment 1b we varied whether the multiple victims were isolated or in a group. We found no significant differences between these two conditions, suggesting that the mere knowledge (by the judge, if not by the victim) that other victims exist was sufficient to produce this effect. Finally, Experiment 1b addressed the possibility that the severity of judgment levied on the perpetrator in the single victim condition could stem from perceiving the harm to a single victim as easier to avoid. In the current experiment, the harmful act was equally avoidable in all conditions, but the negative reactions in the *single victim* condition were still more extreme than in the *multiple victim* conditions.

Our results are consistent with those reported in Nordgren and McDonnell (2010), who found that a hypothetical fraud was judged as worse (and deserving of more severe punishment) when it affected a smaller number of victims. Nordgren and McDonnell (2010) explained their findings as the result of greater victim identifiability in the single victim condition. In the current experiment, however, all the victims were equally anonymous, but participants seemed to have judged the fact that the single victim was unique to accrue the harm suffered. In the following experiment, we kept all victims equally identified to the judges, by eliciting judgments from the victims themselves.

## Experiment 2

Experiment 2 sought to generalize the effect observed in the first two experiments in two ways. Whereas Experiments 1a and 1b examined participants’ intuitions regarding the harm suffered by others, the present experiment tested participants’ actual experiences as victims. Participants experienced situations where they learned they had received ostensibly professional (but false) feedback on their performance on a cognitive task. We varied the number of affected individuals between groups and investigated whether such personally experienced harm would also be perceived as more benign and as less immoral when they were not the only ones subjected to it.

Experiment 2 also provided an additional test of the identifiability account for the findings described above. Victims can be identifiable or unidentifiable when judged by others, but not when they are the target of their own assessments. Thus, if identifiability is the underlying mechanism of the perverse effect of the number of victims on moral judgment and emotional involvement, this effect should not be observed in the victims’ reactions to the harm each of them experiences.

### Method

#### Participants

Eighty-one undergraduate students at an Israeli university (51 Female; mean age = 29.8 years, *SD* = 7.32) volunteered to participate in an online experiment. The participants signed an online consent form at the beginning of the experiment, and were debriefed at the end.

#### Procedure

Participants were randomly assigned to conditions in a 2 (feedback type: benign vs. harmful) by 2 (target of deception: individual vs. multiple) between-subjects design. After providing general demographic information, participants completed a modified version of the Cognitive Reflection Test (CRT; Frederick, 2005) followed by a 10-item personality test (TIPI; Gosling et al., 2003) translated into Hebrew.

Next, participants received (false) feedback, ostensibly computed online based on their performance on the CRT. In the *benign feedback* condition, participants were told that their performance was two standard deviations above the mean of the entire sample, whereas those in the *harmful feedback* condition were told that their performance was two standard deviations below the mean. Following a short filler task (presented as “a complementary test aimed to validate the results of the first analytical test”), participants were given a disclaimer about the deception. Half of the participants (the *multiple victim* condition) were told that they, as well as all participants in the experiment, had received false feedback in the previous stage. The other half (the *individual victim* condition) were told that the decision to deceive them was based on their score on the TIPI test. Finally, participants were asked to rate the morality of the false feedback given by the researchers, and to indicate the extent to which they felt hurt, angry, disappointed, embarrassed, betrayed and/or humiliated by what happened (both were measured on a 0–5 scale).

### Results

#### Perceived Immorality

A 2 (feedback type: benign vs. harmful) by 2 (target of deception: individual vs. multiple) ANOVA revealed a main effect for the target of deception [*F*(1,77) = 4.03, *p* = 0.047, $\eta_{p}^{2}$ = 0.05]. As predicted, participants in the individual target condition rated the act as significantly more immoral (*M* = 2.87, *SD* = 1.26) than those who were told that all the other participants were treated as they had been (*M* = 2.30, *SD* = 1.34). The other effects – feedback type and interaction – were non-significant. **Table 4** presents the means and standard deviations for all four conditions.

**Table 4:** Means (SD’s) of perceived immorality of the act as a function of the feedback type and the deception target in Experiment 2.

Target of deception / Feedback	Benign	Harmful
Individual	2.94 (1.39)	2.81 (1.17)
Multiple	2.40 (1.53)	2.17 (1.04)


#### Negative Feelings

Participants’ reported negative feelings after receiving the disclaimer regarding the deception had high inter-item reliability (Cronbach’s α = 0.81). Therefore, we calculated an index based on the average of these six items, which served as the target for the following analyses.

The 2 (feedback type: benign vs. harmful) by 2 (target of deception: individual vs. multiple) ANOVA indicated a significant interaction between feedback type and the target of deception [*F*(1,77) = 4.11, *p* = 0.046, $\eta_{p}^{2}$ = 0.05]. Simple effects tests showed that among participants in the harmful feedback condition, those who believed they were deceived based on their personal traits rated their negative feelings as more intense [*M* = 1.60, *SD* = 0.83] than those who believed others were also treated the same way [*M* = 1.18, *SD* = 0.29]. However, among those who received the benign feedback, there was no difference in the extent of negative feelings between the two conditions of target of deception (*F* < 1). Moreover, the means indicated an opposite trend, such that negative feeling ratings were lower for participants who believed they were the only ones who had been deceived than those who were told that all the others were treated as they had been (see **Table 5**). Finally, although 63% of our participants were female, we did not find evidence for gender differences that could account for the results.

**Table 5:** Expressed negative feelings (Means and SD’s) a function of the feedback type and the deception target in Experiment 2.

Target of deception / Feedback	Benign	Harmful
Individual	1.33 (0.43)	1.60 (0.83)
Multiple	1.49 (0.72)	1.18 (0.29)


### Discussion

Experiment 2 found that the perverse effect of additional victims on the judgment of a transgression was exhibited not only by non-involved observers or readers of hypothetical scenarios, but also by the actual victims of a real, albeit not very severe transgression. Participants who had been deceived judged the deception as less immoral and less harmful to them when they were told that other participants had also been deceived, relative to when they thought they were the only victims of deception. Interestingly, participants assessed the deception of an individual victim as more immoral, regardless of whether the feedback was benign or harmful. However, their emotional reports portrayed a different picture. Whereas participants in the harmful feedback condition reported greater negative feelings when the feedback was individually targeted, those who were deceived in a self-serving manner showed quite similar levels of negative feelings, whether the deception they experienced was individually oriented or not.

These findings suggest that participants may have perceived the mere existence of others as helpful in alleviating their pain, which is consistent with the beliefs conveyed by the readers of the hypothetical scenarios in Experiments 1a and 1b. Our findings challenge the association between the number of victims and their perceived identifiability as a necessary condition for this effect, since the judges of the transgression in this experiment were also the victims and their own identifiability was equally high in all conditions.

In this experiment, participants experienced actual harm characterized by one of two degrees of severity. Some received false feedback that was positive before learning that it was deceitful, whereas others were given false feedback suggesting they were inferior to their peers. Note, however, that we measured participants’ reactions after we informed them not only about having been deceived but also about the content of the deception and the truth; i.e., that there was no information suggesting they were either superior or inferior to others in the measures we collected. This disclaimer may have evoked qualitatively different emotional reactions, which may have accounted in part for participants’ subsequent responses. In the impersonal conditions, those who were informed that they were not “cognitively inferior” may have felt relieved, whereas those who were informed that their “superiority” was in fact a product of deceit may have felt disappointment. In the personal condition, however, the negative reaction matched the valence of the feedback, even after participants were informed that this feedback was false. This might explain the differences between the patterns we observed in moral judgments and those that were observed in the participants’ emotional reactions.

## General Discussion

The present work replicates and extends findings on the effect of the number of victims on harm perception and moral judgment. Our findings seem to indicate a bias in moral judgment and in assessment of harm such that a transgression affecting several individuals was paradoxically judged as *less* (rather than more) severe and immoral than one affecting a single victim. This was exhibited not only by participants who judged the harmful act from an external point of view, but also when they were the victims themselves. The effect was independent of the physical presence of other victims, or the individual’s ability to communicate with them. Rather, simply knowing that other victims exist reduced reported suffering as well as the extent to which they judged the perpetrator’s behavior as immoral.

Our findings are relevant to studies on helping behaviors (e.g., Kogut and Ritov, 2005; Slovic, 2007) and the relationship between the number of individuals in need of help and the willingness to offer it. These studies have found that the willingness to offer help or a monetary donation sharply decreased as the number of individuals in need increased. However, whereas these works have focused on people’s decisions about whether and to what extent to actively help the victims, the bias we found did not depend on any expectation that the participants would take action.

Our findings challenge the association between the number of victims and their perceived identifiability as a necessary condition for this effect. In Experiment 2, we elicited the victims’ reactions to the transgression committed against them. These victims were obviously identifiable to themselves. Nonetheless, we found that the effect persisted in both cases such that the harm suffered by a lone victim was judged as more severe than the harm suffered by one of several victims.

### Future Directions and Limitations

Our findings provide preliminary evidence for a bias in moral judgment in which the mere existence of other victims reduces assessments of the harm suffered by each harmed individual. This bias was replicated in our three studies, but nonetheless is still subject to several different explanations and boundary conditions. For example, knowing of the existence of many more victims may trigger a dispositional attribution. According to Attribution Theory (Kelley, 1973), an outcome is characterized by low consensus when it is observed on the focal person but not on others. Low consensus increases the extent to which the outcome is attributed to the person’s character or skill rather than to external factors. Judges of the single victim may have construed the fact that no other person was harmed to mean that the victim was somewhat responsible for the outcome; conversely, the presence of other victims may have increased the perceived consensus of their suffering, which in turn could have increased the external attribution of this outcome.

Another possible explanation is that individual and multiple victims provide different reference points for judgments. Because harm suffered by a victim is very difficult to perceive in absolute terms, people may resort to comparative processes to assess this harm. With no obvious reference point against which this harm can be measured, they may compare a victim’s state to her state before the act or to that of society in general (Festinger, 1954). In contrast, the other concrete victims of the same act provide readily available reference points for such comparisons. The judge realizes that the victim’s pain is no worse than that suffered her peers, and this result in lower assessment of each victim’s pain. This account suggests that people’s moral judgments are determined by the available reference points rather than by the objective level of harm caused by the act.

A third explanation stems from the human tendency to think heuristically. People may apply assumptions to their judgments, such as that the existence of other victims has actual benefits, e.g., moral support or cooperation. People might also make a calculation where the magnitude of the harm is “distributed” across several recipients. In some cases, there could be a sound reason for the reduction in the severity of moral judgment when multiple victims are concerned. Nevertheless, as our experiments demonstrate, people might overgeneralize their intuitive rules to situations where the existence of other victims cannot provide any alleviation of the individual victim’s pain.

Finally, it is worth noting that the scope of the current study was limited to relatively mild harm, suffered by few individuals. This raises questions as to whether the same mechanism can be generalized to cases where harm that is more significant is evoked on much larger scales. The recent Volkswagen emission cheating scandal that was widely covered in the media can serve as a recent example. About 500,000 owners were affected in the US, compared to 11 million across the rest of the world. Would Americans have judged the acts of the VW cooperation more severely had fewer victims been affected? Such important questions call for future studies that investigate moral judgments in case studies from the field, especially ones involving large-scale harm.

## Conclusion

As long as the legal system incorporates the magnitude of harm committed by transgressors in the evaluation of punishment, fair treatment by the law requires accurate appraisal of harm. Our findings show that intuitive assessments of harm and immorality are prone to a systematic bias in which the harm done to each of many victims is often judged less severely than harm done to one victim. This bias is shared by third-party judges and victims alike. Overcoming this bias may help to maintain a more balanced system of assessing punishment, entitlement, and retribution. Until then, victims’ only consolation is that their misery loves company.

## Author Contributions

SA, UH, and DK developed the study concept; DK, UH, and KS developed the stimuli and designed the experiment; DK and UH collected the data; DK and KS analyzed and interpreted the data; DK, UH, and SA wrote the article.

## Conflict of Interest Statement

The authors declare that the research was conducted in the absence of any commercial or financial relationships that could be construed as a potential conflict of interest.
